# QUERI and implementation research: Emerging from adolescence into adulthood: QUERI Series

**DOI:** 10.1186/1748-5908-4-12

**Published:** 2009-03-06

**Authors:** David Atkins

**Affiliations:** 1 VA Quality Enhancement Research Initiative, Veterans Health Administration, Washington, DC, USA

## Abstract

The Quality Enhancement Research Initiative (QUERI) program and implementation research have both come of age in the 10 years since QUERI was established. Looking forward, if QUERI and the field of implementation science are to mature successfully, we will need to address a series of challenges. First, we need to more clearly demonstrate how applying principles of implementation science leads to more effective implementation and communicate those lessons to our partners and funders. Second, we will need to engage in the ongoing debate over methodological standards in quality improvement and implementation research. Third, a program like QUERI needs to become more relevant to the daily decisions of key stakeholders. Fourth, if we hope to sustain interest in implementation science, we will need to demonstrate the business case for more effective implementation. Fifth, we need to think creatively about how to nurture the next generations of implementation researchers and front-line "connectors," who are critical for accelerating implementation. Finally, we need to strengthen the connections between implementation research and the other operational and research activities that influence change in healthcare systems.

The excitement of entering adulthood is tempered by the challenge of new responsibilities and expectations. What is essential is that we continue to learn and move forward. For implementation science and for QUERI, the next decade looks to be one filled with exciting possibilities, new partnerships, increasing relevance, and real accomplishment.

## Background

The Quality Enhancement Research Initiative (QUERI) program and implementation research have come of age together since 1998 when QUERI was launched as part of a set of sweeping changes occurring within the Veterans Health Administration (VA) [1]. The VA's transformation [2-4] – which involved regionalizing care, increasing performance measurement and accountability, expanding use of health information technology, and emphasizing evidence-based practices and primary care – was based on proven principles of change in large organizations, but our understanding of how to speed the adoption of effective healthcare across a large organization was still in its infancy. QUERI's tenth birthday offers a timely opportunity to take stock of how much QUERI and implementation science have grown up in the intervening decade, and to reflect on the opportunities and challenges we can foresee over the next ten years [4].

## Discussion

Adulthood is typically marked by some predictable milestones: developing an identity distinct from our parents, becoming self-sufficient, becoming competent in a career, and recognizing one's responsibility to others. Against these benchmarks, both QUERI and implementation science have clearly emerged from early adolescence. Within the VA, QUERI has developed a distinct identity from its "parent" – health services research [4]. The support for QUERI has both increased and broadened, with increases in funding and greater participation in QUERI from various parts of the VA healthcare delivery system. Implementation science also has developed a distinct identity over this time. The well-documented and persistent gap between the care we know to be effective and that which is routinely delivered [5,6], what the Institutes of Medicine called the "quality chasm" [7], has begun to persuade policymakers that traditional basic science and clinical research alone are not sufficient to tackle the big problems facing American healthcare [8]. Funding agencies and foundations in the U.S. have gradually increased attention to "translation research" [9-14], seeking to shorten the 17 years from when an intervention is shown to be effective and when it is widely applied [15]. Finally, implementation researchers in QUERI and elsewhere have demonstrated "competence" to their colleagues in health services and clinical research by producing a growing body of sophisticated, rigorous and useful studies of the implementation process, as indicated in the preceding reports in this series [4].

A more challenging standard for assessing our progress is to compare it to a benchmark of success in clinical medicine, such as the evolution of the "cholesterol hypothesis." Epidemiologic studies identified risk factors for heart disease which were pursued through basic science studies, elucidating the basic pathophysiology of heart disease and the cellular mechanisms of cholesterol metabolism. This opened the way for more effective and targeted interventions, which in turn were tested in a progressive series of trials. Studies demonstrating efficacy of behavioral and pharmacologic interventions against intermediate outcomes, such as lipid levels and atherosclerosis, were followed by large multi-center trials showing effectiveness in reducing morbidity and mortality. Finally, economic studies demonstrated that cholesterol reduction was cost-effective, and clinical and public health initiatives were launched to improve the population-wide delivery of these interventions.

This admittedly high standard illustrates some of the ground we have yet to cover. Both implementation research and QUERI have produced a wealth of studies describing the epidemiology of healthcare delivery and variations in quality. Researchers have developed more sophisticated methods to describe the process of implementation and quality improvement [16-18]. We have begun to advance our basic understanding of the process of improving healthcare – breaking open the "black box" to understand the contextual factors, barriers, facilitators, mediators, and moderators that underlie successful implementation – and to develop models that describe the critical pathways of the improvement process.

However, we must do a better job of applying this knowledge consistently to devise and empirically test our implementation interventions. The strategies tested in implementation research at times resemble applying broad spectrum antibiotics to a patient with an unknown source of fever, rather than carefully selecting therapy based on the source of infection, susceptibility, and relative costs and safety. As QUERI has matured, investigators more consistently incorporated implementation theory and implementation science goals into their individual projects and strategic plans. More rigorous empiric studies of implementation have multiplied and produced notable successes both within QUERI and elsewhere, but these have largely been in process improvements (better delivery of effective care, or Steps 4–5 in the QUERI process) rather than the health outcomes that mark the final step of the QUERI process (see Table 1). Finally, we have yet to see our accumulated knowledge about implementation routinely integrated into efforts to improve the organization of healthcare systems.

**Table 1:** The VA Quality Enhancement Research Initiative (QUERI)

The U.S. Department of Veterans Affairs' (VA) Quality Enhancement Research Initiative (QUERI) was launched in 1998. QUERI was designed to harness VA's health services research expertise and resources in an ongoing system-wide effort to improve the performance of the VA healthcare system and, thus, quality of care for veterans.

QUERI researchers collaborate with VA policy and practice leaders, clinicians, and operations staff to implement appropriate evidence-based practices into routine clinical care. They work within distinct disease- or condition-specific QUERI Centers and utilize a standard six-step process:
1) Identify high-risk/high-volume diseases or problems.
2) Identify best practices.
3) Define existing practice patterns and outcomes across the VA and current variation from best practices.
4) Identify and implement interventions to promote best practices.
5) Document that best practices improve outcomes.
6) Document that outcomes are associated with improved health-related quality of life.

Within Step 4, QUERI implementation efforts generally follow a sequence of four phases to enable the refinement and spread of effective and sustainable implementation programs across multiple VA medical centers and clinics. The phases include:
1) Single-site pilot,
2) Small-scale, multi-site implementation trial,
3) Large-scale, multi-region implementation trial, and
4) System-wide rollout.

Researchers employ additional QUERI frameworks and tools, as highlighted in this *Series*, to enhance achievement of each project's quality improvement and implementation science goals.

Looking forward, if QUERI and the field of implementation science are to grow to be fully successful adults, we will need to address a series of challenges.

First, we need to do a better job of tying implementation science to more effective implementation. This means going beyond "basic discovery" about the implementation process to demonstrating and communicating how these insights produce better interventions and more rapid improvements. We now have a number of theoretical implementation models that enumerate specific aspects of the implementation process and identify critical mediators and moderators of success [19-25]. A more compelling test of their value is to show that implementation strategies based on these models are more effective or efficient than those developed without them. A limited number of such studies have been published [26], and QUERI investigators are now developing proposals to directly compare enhanced theory-based strategies to more traditional methods used by the VA healthcare system for implementing new programs.

Second, QUERI and implementation research will need to engage thoughtfully in the debate now playing out over methodological standards in quality improvement and implementation research [27-30]. Each side in this debate has been guilty of caricaturizing the position of their opponent, making it appear that one side rejects any study that is not a randomized clinical trail, while the other will accept any evidence that supports their favorite quality improvement intervention. In reality there is a serious discussion to be had over how we ensure that evidence is both valid and applicable, and how we balance our desire to foster timely improvements in care with the need to protect against promoting ineffective or even harmful changes. This requires considering a set of distinct but related questions: What can and can't we learn about the change process from a more diverse set of studies (including qualitative studies)? What are the important sources of bias in different non-randomized designs, how do they vary with the intervention and setting, and how can they be reduced? How do we determine when randomized studies of complex systems are applicable and to whom? And, how do we decide when we have "sufficient" evidence for promoting changes in practice at different levels of the health care system?

Third, QUERI and implementation research needs to become more relevant to the daily decisions of our key stakeholders. While research cannot be responsive to every need of managers and policymakers, we need to understand their priorities and their constraints. There will always be a healthy tension between the imperatives of research – emphasizing rigor and high certainty at the expense of longer timelines – and those of managers, who prefer answers that are timely and "good enough." Increasing our relevance means aligning the priorities of research and the healthcare system as early as possible [31,32].

Although QUERI has a unique advantage in being embedded in a working healthcare system, with a defined audience of VA managers and policymakers, we often cannot provide them as definitive answers as they would like, nor as soon as they require. However, implementation research can help them understand the tradeoffs and uncertainty involved as they consider ways to roll out new programs. Since only a minority of new programs being implemented in the VA and elsewhere have evolved through a progressive, empirical process advocated by implementation science, we need to devise better ways to learn from the real-world "experiments" being conducted in healthcare systems. Closer and earlier partnerships between researchers and managers may allow us to generate more robust evidence from more varied settings about what makes implementation successful.

Fourth, sustaining interest in implementation science will require demonstrating the business case for more effective implementation. Careful cost analyses of individual components of the implementation process may help us design more cost-effective strategies. The QUERI program explicitly promotes economic analyses within its studies, but we may need to align our economic models more closely with the budgeting and decision-making processes at different levels – from individual practices to medical centers to larger networks. The successful uptake and sustainability of implementation interventions will depend on being able to show that they provide good "value" from the perspectives of different decision-makers.

Fifth, we need to examine how to nurture the next generations of both the implementation researchers and the front-line "connectors" who are critical for accelerating implementation. Programs such as QUERI, the National Institutes of Health Clinical and Translational Science Award (CTSA) program, and initiatives from major foundations have helped create new career paths for implementation researchers, as has the emergence of journals such as *Implementation Science *where these investigators can publish peer-reviewed research. More challenging is developing and sustaining the expertise of the non-academic facilitators who understand the improvement process and the specific context of the practice or institution in which they are trying to institute change. We need to determine the optimal mechanisms for training and retaining these critical "change agents," which may require cooperation between those in charge of research, healthcare delivery, and quality.

Sixth, QUERI and implementation research in general need to strengthen the connections to the other operational and research activities that influence change in healthcare systems. These include existing quality measurement and improvement activities, post-graduate and continuing education, and health informatics [33]. Our implementation efforts will always be working uphill if they are not aligned with the priorities reflected in existing performance measures and incentives, educational programs, and health information systems. Implementation research has much to offer to these disciplines, and they, in turn, are often critical components of implementation interventions. Working together we can multiply the power of our individual resources to achieve meaningful change.

Seventh, we probably need to engage in some "expectations management." The rewards of better implementation are tantalizing (How can it be that only half of effective care is routinely delivered?), but the reality is that change is hard and the learning can be slow. At present, we are investing pennies on implementation research for every dollar spent on basic and clinical research and for every $100 spent on healthcare. We need to be realistic about what success will look like and transparent about our goals and objectives. In QUERI, we need to balance the temptation to extend our work into new areas with new partners, with the desire to make more substantive progress on high-priority objectives.

This leads to the final and most important challenge for implementation research – to demonstrate impacts on healthcare and health that are meaningful to our stakeholders. John Eisenberg, the late Director of the Agency for Healthcare Research and Quality (AHRQ), suggested that all health services researchers should strive to answer the "Porter question" [34]. Congressman John Porter, a strong advocate for research, had asked him "So, what difference have you made?" What Porter meant was that the traditional measures of a successful research program (e.g., grants funded, investigators trained, results published, careers advanced) were no longer sufficient for a field premised on the need to tackle pressing "real-world" healthcare problems. We need to challenge ourselves to produce measurable improvements for healthcare and for the patients we serve. This is no easy task, given the size and complexity of the systems and processes with which we work, and it won't come quickly. But it is a challenge that can be met if we work with the right partners and leverage our efforts effectively. If we can meet this responsibility to the patients and healthcare systems we study, we will have become truly adult.

## Summary

Becoming an adult is both exciting and anxiety-provoking. The excitement of new possibilities is tempered by the challenge of new responsibilities and expectations. It is just as important, however, to recognize that reaching adulthood is only the beginning of a lifelong process filled with failures as well as successes. What is essential is that we continue to learn and move forward. For implementation science and for QUERI, the next decade looks to be one filled with exciting possibilities, new partnerships, increasing relevance, and real accomplishment. Our parents should be proud.

## Competing interests

The author declares that he has no competing interests.

## Authors' contributions

DA conceived of and wrote this article.

## Disclaimer

The views expressed in this article are those of the author, who is responsible for its contents, and do not necessarily represent the views of the U.S. Department of Veterans Affairs.
